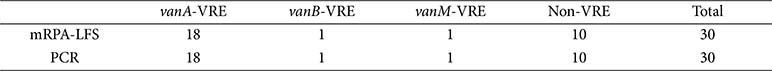# Simultaneous and Accurate Visual Detection of Vancomycin-Resistant Enterococci *vanA*, *vanB* and *vanM* by Multiplex Recombinase Polymerase Amplification Combined with Lateral Flow Strip

**DOI:** 10.4014/jmb.2508.08037

**Published:** 2025-11-19

**Authors:** Yuqing Xing, Tingting Hu, Siyi Zhou, Jilu Shen

**Affiliations:** 1 Department of Clinical Laboratory, The First Affiliated Hospital of Anhui Medical University, Hefei 230022, P.R. China; 2 Department of Clinical Laboratory, Anhui Public Health Clinical Center, Hefei 230012, P.R. China

**Keywords:** Vancomycin-resistant enterococci, recombinase polymerase amplification, lateral flow strips, rapid detection

## Abstract

Vancomycin-resistant *Enterococcus* (VRE) has demonstrated increasing global prevalence in recent years. Clinical detection currently relies on phenotypic methods including agar screening, minimum inhibitory concentration (MIC) testing, Kirby-Bauer disk diffusion, and Etest. In addition, molecular approaches such as polymerase chain reaction (PCR) and quantitative PCR (qPCR) can be applied for VRE identification. Nevertheless, these methods cannot achieve point-of-care detection (POCT). Thus, novel rapid diagnostic platforms have become urgently needed for curbing VRE transmission and containing nosocomial outbreaks. Recombinase polymerase amplification (RPA) and lateral flow strips (LFS) are effective tools for achieving rapid POCT. In this study, RPA was combined with LFS to establish a fast, sensitive, and specific detection method. This study established a multiplex RPA-LFS (mRPA-LFS) that delivers results within 30-40 min, with detection limits of 10^2^ copies/μl for *vanA*, *vanB*, and *vanM*. Notably, the assay demonstrated high specificity without cross-reactivity to common bacterial/fungal pathogens, and showed 100% concordance with conventional PCR in 30 clinical samples. In this study, a rapid detection assay for *vanA*, *vanB*, and *vanM* genes in VRE was developed using mRPA-LFS technology. Characterized by high sensitivity, specificity, operational simplicity, and cost-effectiveness, this method is suitable for on-site detection.

## Introduction

Enterococci are Gram-positive bacteria, with 58 species identified to date. Among these, *Enterococcus faecium* and *Enterococcus faecalis* cause the majority of clinical infections [1]. These microorganisms are widely distributed in the gastrointestinal tracts of humans and animals, and may also be detected in feces-contaminated water, soil, plants, and food [2]. Under specific conditions, enterococci may colonize oral and vaginal mucosal surfaces [3]. Although typically constituents of the gut microbiota, enterococci can act as opportunistic pathogens when host barrier function is compromised, causing serious infections including urinary tract infections, intra-abdominal infections, bacteremia, endocarditis, and meningitis [4].Vancomycin, a glycopeptide antibiotic exhibiting potent activity against numerous Gram-positive bacteria, is utilized for treating infections caused by ampicillin-resistant enterococci (ARE) and high-level aminoglycoside-resistant enterococci (HLARE) [5]. Since the initial identification of vancomycin-resistant enterococci (VRE) in the 1980s, their global prevalence has steadily increased. This trend has substantially reduced clinicians' therapeutic options for enterococcal infections, posing a significant threat to public health [6-8]. Annually, VRE infections are estimated to cause at least 5,400 deaths and incur over $500 million in additional healthcare costs [9]. Moreover, the mortality rate of bloodstream infections caused by VRE exceeds that of infections caused by vancomycin-susceptible enterococci (VSE) [10].

Resistance in VRE is primarily mediated by van gene clusters. These clusters encode functional proteins that catalyze the synthesis of altered peptidoglycan precursors with significantly reduced vancomycin affinity , thereby reducing drug-target binding and conferring resistance [11]. Nine van cluster types have been identified: *vanA*, *vanB*, *vanC*, *vanD*, *vanE*, *vanG*, *vanL*, *vanM*, and *vanN*, with *vanA*, *vanB*, and *vanM* being most prevalent clinically [12]. VRE genotypes vary significantly in their resistance levels, inducibility, and cross-resistance patterns to glycopeptide antibiotics. The *vanA* genotype is the most prevalent and clinically significant, conferring high-level, inducible resistance to both vancomycin and teicoplanin. This genotype can spread horizontally via plasmids or transposons, facilitating the rapid dissemination of resistance and posing a major challenge for infection control in healthcare settings. The *vanB* gene cluster confers variable levels of inducible resistance to vancomycin but retains susceptibility to teicoplanin [13]. Although typically chromosomal, *vanB* can also be plasmid-borne, and its prevalence has increased in some European countries in recent years [14]. The *vanM* gene cluster was first identified in Shanghai, China, in 2006 and has since been reported in other regions, including Singapore [15]. Similar to *vanA*, the *vanM* genotype confers high-level, inducible resistance and has become the second most prevalent genotype among VRE isolates in China [16]. Partial deletions within van gene clusters can lead to functional inactivation and phenotypic silencing. These silent resistance clusters persist in the bacterial genome and may regain resistance through genetic recombination, posing a risk of being undetected by phenotypic methods alone. Therefore, rapid detection of vancomycin resistance genes is critical for precision medicine, infection control, and public health management. Current phenotypic detection methods for VRE include agar screening, broth microdilution (MIC), Kirby-Bauer disk diffusion, and Etest [17]. However, these approaches are time-intensive, exhibit limited sensitivity, and cannot directly identify resistance genotypes. Although polymerase chain reaction (PCR) has been employed for VRE strain identification for decades [17, 18], conventional PCR protocols remain technically complex due to required gel electrophoresis, limiting their utility for rapid point-of-care testing (POCT).

Isothermal amplification-based molecular diagnostics have advanced significantly in recent years, offering rapid analysis, portability, and minimal instrumentation requirements. Recombinase polymerase amplification (RPA), developed in 2006, amplifies target genes at constant temperatures (37–42°C) without precision instruments. This technology demonstrates substantial potential for POCT and serves as an efficient complement to conventional PCR [19]. RPA amplicons can be detected via multiple methods including agarose gel electrophoresis, real-time fluorescence quantification, or electrochemical detection. Alternatively, lateral flow strips (LFS) can be used to visualize the amplicons in 5 min, which is simple, cost-effective and highly practical [20-22]. RPA-LFS has been successfully used for the molecular diagnosis of diseases caused by pathogenic bacteria, including influenza A/B virus dual-gene detection [23]. In this study, we developed a multiplex RPA-LFS (mRPA-LFS) platform using end-modified primers specific to *vanA*, *vanB*, and *vanM* genes. In contrast to conventional methods that require multiple hapten-antibody pairs for target discrimination, this study introduces a novel strategy employing a universal hapten (FITC) to label all forward primers, while utilizing unique nucleic acid tag (NAT) sequences on the 5' end of reverse primers for parallel signal identification. This approach simplifies the labeling procedure, reduces costs, and, most significantly, greatly enhances the multiplexing capability and scalability of the assay. Consequently, a simple, specific, and sensitive method was established, providing a technical foundation for the rapid detection of clinically relevant VRE resistance genotypes.

## Materials and Methods

### Reagents and Chemicals

All the primers and capture probes used in this study were independently designed by our laboratory and synthesized by Shanghai Sangon Biotech Company (China). The RPA basic amplification kit was purchased from AmpFuture Biotech (China); The RPA product purification kit was purchased from Sparkjade (China); MIC Test Strip was purchased from Liofilchem (Italy); Lysis Buffer for Microorganism to Direct PCR was purchased from Takara Bio (China); 10×PBS buffer, agarose and proteinase K solution (20 mg/mL) were all obtained from Sangon Biotech; Small molecule DNA marker, 6×Loading buffer, 4S Red plus nucleic acid dye (1000×) were purchased from YEASEN (China); Sample pads, conjugate pads, nitrocellulose membranes (NC membranes), polyvinyl chloride adhesive backing pads, and absorbent pads were from the Jie-Ning Biotech. Co. Ltd. (China); All other common chemical reagents of analytical grade were purchased from Sinopharm Chemical Reagent Co., Ltd.(China).

### Clinical Specimens and Source of the Strain

The strains used in this study included strains preserved in the laboratory, strains donated by Huashan Hospital affiliated to Fudan University, and strains isolated from clinical samples. All strains were identified by MALDI-TOF MS mass spectrometer. A total of 30 clinical enterococcus strains were collected, including 20 VRE strains and 10 non-VRE strains. To verify the specificity of this method, 12 samples of other common pathogens (*Escherichia coli*, *Klebsiella pneumoniae*, *Acinetobacter baumannii*, *Streptococcus pneumoniae*, *Staphylococcus aureus*, *Staphylococcus epidermidis*, *Pseudomonas aeruginosa*, *Candida albicans*, *Bacillus cereus*, *Campylobacter jejuni*, *Shigella sonnei*, *Salmonella typhi*) were collected. In addition, we constructed *E. coli* strains containing standard plasmids carrying *vanA*, *vanB*, and *vanM* genes, which were used as reference strains for analyzing the sensitivity.

### Antimicrobial Susceptibility Testing

Thirty clinical isolates were streaked onto blood agar and incubated at 37°C for 24 h. A bacterial suspension equivalent to a 0.5 McFarland standard was prepared from fresh cultures. This suspension was then evenly swabbed onto Mueller-Hinton (M-H) agar plates in three directions. After the inoculum had dried, pre-warmed Etest strips were applied to the agar surface within 15 min and were not moved after placement. The plates were incubated inverted at 35°C for 18–24 h. The minimum inhibitory concentration (MIC) was read for each isolate, and the results are provided in Supplementary Material 1.According to CLSI guidelines, the vancomycin susceptibility breakpoints for enterococci are as follows: susceptible (S), MIC ≤4 μg/ml; intermediate (I), MIC 8–16 μg/ml; and resistant (R), MIC ≥32 μg/ml. Based on these criteria, the testing identified 20 resistant strains and 10 susceptible strains.

### Nucleic Acid Extraction

Nucleic acid was extracted from 30 clinical strains of *Enterococcus* according to the instructions of the Lysis Buffer for Microorganism to Direct PCR and stored at -80°C. The DNA concentrations of the analyzed clinical samples are provided in Supplementary Material 1. The specific operation was as follows: 50 μl of Lysis Buffer for Microorganisms to Direct PCR was pipetted into 1.5-ml Eppendorf tube. Next, a single colony was collected using a sterile cotton swab and placed in 1.5-ml Eppendorf tube. Subsequently, it was thermally denatured at 80°C for 15 min and centrifuged at low speed. Finally, 2 μl of the lysed supernatant was taken as a template for the subsequent reaction. As shown in Table 1, nucleic acids of 14 other pathogens were also extracted.

### Design of Primers and Probes

The sequences of *vanA*, *vanB* and *vanM* genes were downloaded from the official website of NCBI (https://www.ncbi.nlm.nih.gov/genbank/), and highly conserved sequences were obtained by comparing multiple sequences using SnapGene software (version 6.0.2; Dotmatics, USA). According to the conserved sequence, multiple pairs of RPA primers were designed using Primer Premier 5 software (PREMIER Biosoft, USA). The NCBI-BLAST online tool was employed to perform species-specific validation of the designed primers. Subsequently, optimal primers were selected for modification. Among them, the 5 'ends of the three forward primers were all labeled with FITC, and the 5' ends of the three reverse primers were functionalized through conjugation to orthogonal NAT sequences via C12 spacers. Complementary capture probes were designed for each NAT sequence. Finally, the sequence information of the modified forward primers, reverse primers and capture probes is detailed in Table 2.

### LFS Strip Preparation

LFS were prepared according to methods described in previous studies, with modifications implemented [24]. The LFS strip was composed of continuous superposition: sample pad, conjugate pad with gold nanoparticles and FITC antibody (AuNP-Ab), nitrocellulose filter (NC) membrane, adsorption pad as well as backing card. The AuNP were synthesized using sodium citrate tannin reduction, labeled by FITC antibody and sprayed onto conjugate pads. The NC membrane was used for immobilizing three test lines (T-lines) and one control line (C line), which were sprayed with three specific biotinylated capture probes (CP_*vanA*_ to *vanA* on T1 line, CP_*vanB*_ to *vanB* on T2 line, and CP_*vanM*_ to *vanM* on T3 line, respectively) and goat anti-mouse secondary antibody (Anti-Ab) on the C-line, respectively. Of note, before the spraying, all the biotinylated probes were mixed with streptavidin. The backing card acts as the substrate to support the assembly of these four components. It should be mentioned that each pad should be overlapped with adjacent pads about 2 mm to ensure the successful migration of the solutions. Test strips should be stored in a cool, dry place away from light at temperatures between 4°C and 30°C. The shelf life is 12 months.

### RPA Reaction Analysis and Multiple RPA Reaction Analysis

Extracted nucleic acid was used as the RPA amplification template, and amplification was performed according to the manufacturer's instructions for the Amp-Future isothermal amplification kit. Briefly, the 50 μl volume for RPA consisted of 29.4 μl of diluted complex aqueous solution, 2 μl of 10 μM forward primer, 2 μl of 10 μM reverse primer, 4.1 μl of template DNA, and 10 μl of ddH2O. This mixture was inserted in a tube containing the lyophilized enzyme component, following which the reaction pellets were resuspended. Afterward, 2.5 μl of 280 nM magnesium acetate was added to the reaction tube cap, and after centrifugation, the reaction mixture was incubated at 37°C for 20 min (after mixing the reaction system, an equivalent amount of paraffin oil was added to the surface to prevent aerosol pollution). Lastly, the RPA product was purified using a RPA product purification kit and analyzed by electrophoresis on a 3% agarose gel.

The optimization of mRPA systems is as follows: dissolve the freeze-dried enzyme pellet of 29.4 μl of A buffer, and then add 1.0 μl of each primer pair of *vanA*, *vanB*, and *vanM*, 2.5 μl of B buffer, 5.1 μl of DNA template, and 7.0 μl of ddH_2_O in sequence.

### Multiple Detection of VRE with Designed LFS

For detection of amplified products, 1 μl RPA amplification product were diluted wit 49 μl running buffer (2×PBS, 1% BSA, 0.5% PEG 20000, pH 7.4). A droplet of 50 μl of diluted sample was then dropped onto the sample pad of the LFS. The T1–T3 lines and C-line intensities were analyzed within 5 min.

### Sensitivity and Specificity Assessment

For sensitivity assessment, recombinant plasmids containing *vanA*, *vanB*, and *vanM* genes served as standard DNA templates. We calculated the initial copy concentration, and prepared six different concentration solutions by serial dilution in a 10-fold gradient:10^6^, 10^5^, 10^4^, 10^3^, 10^2^, 10^1^ copies/μl. These diluted plasmid templates were subsequently used to determine the detection limit of the mRPA-LFS platform. The specificity of the platform was evaluated using the pathogen nucleic acids described in Table 1 as templates for mRPA-LFS analysis.

### Evaluation of Its Application in Clinical Specimen Examination

A total of 21 VRE strains and 9 non-VRE strains were tested using the mRPA-LFS assay, and compared the results with conventional PCR methods. We then calculated the sensitivity and specificity of our platform. The aim was to verify the accuracy and reliability of the new detection method and determine its potential application value in clinical diagnosis. Sensitivity was calculated as 100×number true positive (TP)/ (number TP+ number false negative [FN]), while specificity was calculated as 100× number true negative (TN) / (number TN + number false positive [FP]).

## Results

### Working Principle of the mRPA-LFS Platform

**Fig. 1:**
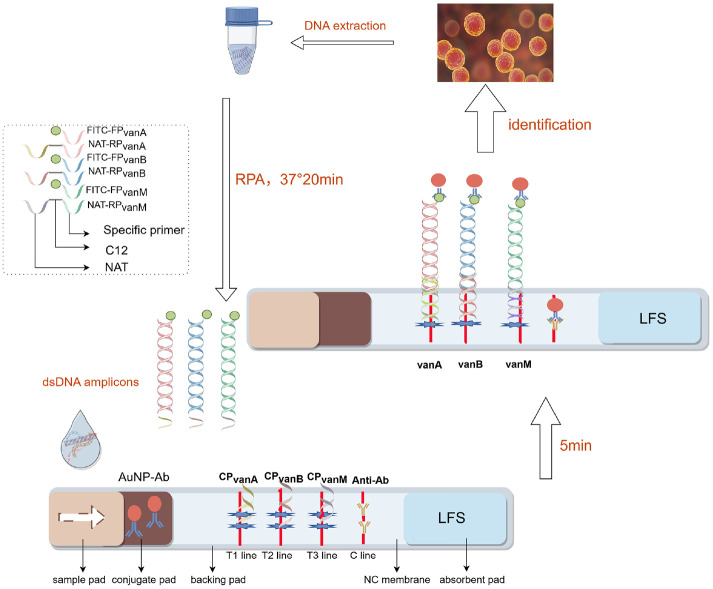
Schematic diagram of mRPA-LFS platform for simultaneously detecting *vanA*,*vanB* and *vanM*.

For building the integrated detection-verification platform of mRPA-LFS, the amplification primers sets are designed according to following rules: the 5' ends of the three forward primers were all labeled with FITC, and the 5' ends of the three reverse primers were functionalized through conjugation to orthogonal NAT sequences via C12 spacers. NAT-RP (NAT-RP*vanA*, NAT-RP*vanB*, and NAT-RP*vanM*) are required to contain three functional regions including a NAT sequence, a C12 spacer for preventing NAT from being polymerized during amplification, and a specific primer sequence for triggering polymerization amplification. Of note, these three NATs of reverse primers have the different sequences, which are complementary to their CPs immobilized on the various T lines of LFS. As shown in Fig. 1, when target DNA is present, the FITC-labeled end of the amplicon binds to the AuNP-Ab conjugate, while the NAT-tagged end hybridizes with the capture probe on the corresponding T-line. The AuNP-Ab is anchored to the T-line through this dsDNA amplicon bridge, turning the line red. Conversely, without target DNA, no dual-labeled dsDNA amplicons are generated, preventing color development on all three T-lines. Regardless of target presence, excess AuNP-Ab conjugate binds to immobilized Anti-Ab on the C-line, causing it to develop color. Based on this principle, mRPA and LFS technologies were integrated, enabling simultaneous, rapid, and sensitive detection of all three VRE resistance genes (*vanA*, *vanB*, *vanM*) with visual naked-eye readout.

### Feasibility Evaluation of the mPRA-LFS

**Fig. 2:**
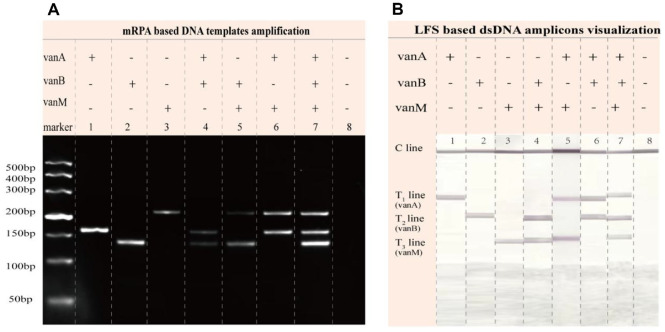
Validation of method feasibility. (**A**) Electrophoresis analysis results: (1) *vanA*, (2) *vanB*, (3) *vanM*, (4) *vanA* *vanB*, (5) *vanB* *vanM*, (6) *vanA* *vanM*, (7) *vanA* *vanB* *vanM*, (8) Analysis template has been replaced with water.; (**B**) LFS analysis results: (1) *vanA*, (2) *vanB*, (3) *vanM*, (4) *vanB* *vanM*, (5) vsnA *vanM*, (6) *vanA* *vanB*, (7) *vanA* *vanB* *vanM*, (8) The analysis template has been replaced with water.

To verify the feasibility of the mRPA-LFS method for simultaneous detection of three drug resistance genes (*vanA*, *vanB*, and *vanM*), electrophoresis and LFS analysis were conducted. Electrophoresis analysis results (Fig. 2A) demonstrated that when only *vanA* (lane 1), *vanB* (lane 2), or *vanM* (lane 3) DNA templates were present, a single characteristic amplification band appeared in the corresponding lane. When DNA templates of two resistance genes coexisted (lanes 4, 5, 6), two corresponding amplification bands were observed. Simultaneous presence of all three resistance gene DNA templates (lane 7) yielded clearly visible bands for all targets. Notably, specific bands only appeared when their corresponding target components were present; when water replaced the analysis template (lane 8), no bands were observed. These results demonstrate that the mRPA system achieves synchronous specific amplification of all three target resistance genes without cross-reactivity. After verifying the mRPA, the feasibility of simultaneous multiple screening with LFS was explored using dsDNA amplicons as the loading samples, which were produced by abovementioned mRPA. As depicted in Fig. 1B, strip 1, 2, and 3 with one T line indicate one kind of dsDNA amplicons. Strip 4, 5, and 6 with two T lines indicate two kinds of dsDNA amplicons. Strip 7 with three T lines shows three amplicons. T1, T2, and T3 lines are ascribed to the dsDNA amplicons of *vanA*, *vanB*, and *vanM*, respectively while strip 8 of control group shows no visible T line. These LFS results are in well agreement with those of mRPA and reveals the excellent availability of LFS. Taken both the mRPA and LFS feasibilities into account, we can surely confirm the availability of the integrated platform of mRPA-LFS for rapid and simultaneous multiple detection.

### Evaluation of Sensitivity and Specificity of the mRPA-LFS Platform

**Fig. 3:**
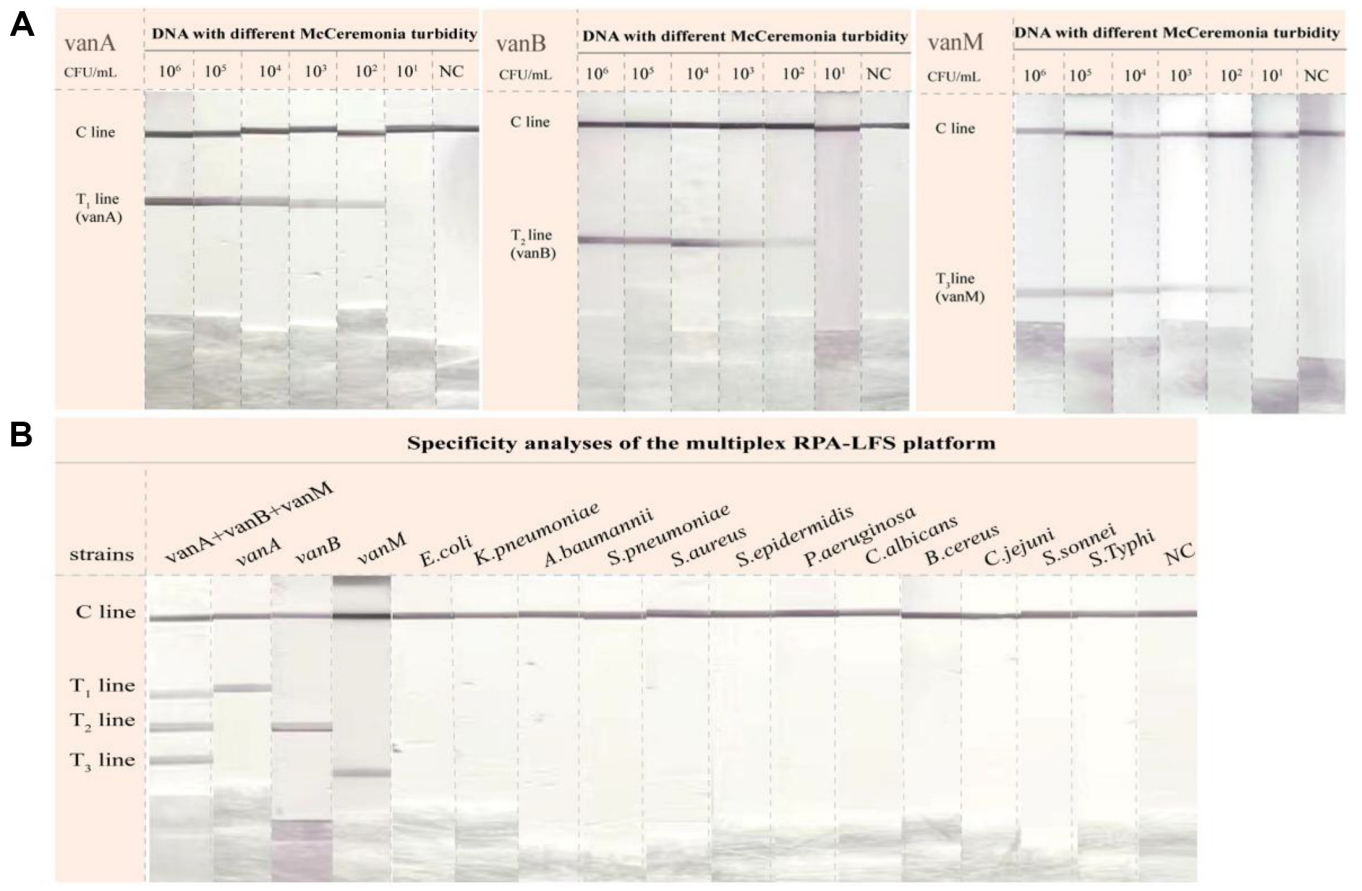
Sensitivity and specificity of the mRPA-LFS platform. (**A**) Sensitivity examination results. (**B**) Specificity examination results.NC indicates that the analysis template has been replaced with water.

To evaluate the lowest detection limit of mRPA-LFS, standard plasmids carrying *vanA*, *vanB*, and *vanM* genes were serially diluted the cloning vector to final concentrations of 10^6^, 10^5^, 10^4^, 10^3^, 10^2^, and 10^1^ copies/μl. As shown in Fig. 3A, the mRPA-LFS detection limits for *vanA*, *vanB*, and *vanM* were as low as 10^2^ copies/μl. We evaluated the specificity of mRPA-LFS using the other pathogen nucleic acids described in Table 1. As shown in Fig. 3B, the T1 line developed red color only in the presence of *vanA*, the T2 line only with *vanB*, and the T3 line exclusively with *vanM*. No color development occurred on test lines when challenged with common pathogens not carrying these three resistance genes. These results demonstrate excellent specificity of the proposed method.

### Clinical Sample Validation

**Fig. 4:**
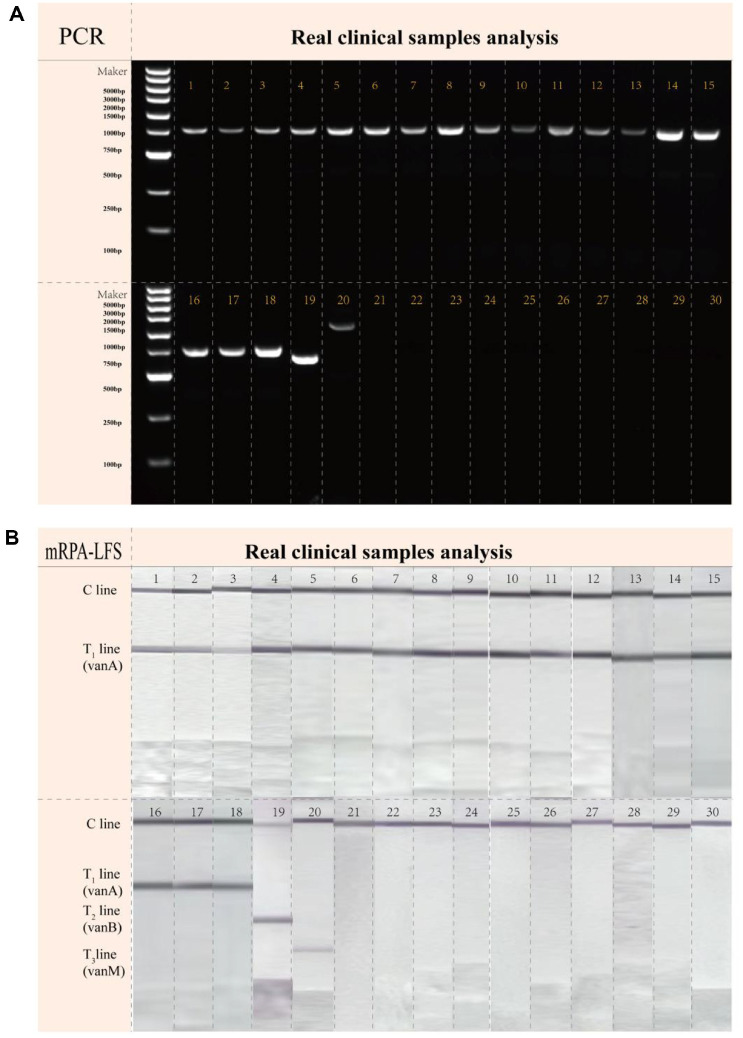
30 clinical strains were tested using the PCR (**A**) and mRPA-LFS (**B**) detection method: strains 1-18 are *vanA*-type VRE, strain 19 is *vanB*-type VRE, strain 20 is *vanM*-type VRE, and strains 21-30 are vancomycin-sensitive enterococci.

In this study, a total of 30 clinical Enterococcus strains were analyzed using both mRPA-LFS and conventional PCR (Fig. 4). The mRPA-LFS method accurately identified 20 VRE and 10 non-VRE strains, with complete concordance in resistance genotyping compared to PCR results (Table 3). The sensitivity, specificity, and concordance rate of this research platform were 100.0%.

## Discussion

Vancomycin has served as a last-resort treatment for multidrug-resistant enterococcal infections since its introduction in the 1950s [25]. However, the emergence of VRE has posed a significant challenge to clinical management. Vancomycin resistance in enterococci is primarily mediated by genetic mutations or the acquisition of resistance genes, which are disseminated among bacterial populations through horizontal gene transfer [26]. Other risk factors for VRE infections include prolonged antibiotic exposure, immunocompromised status, and a history of healthcare exposure [27]. The global prevalence of VRE has risen substantially in recent years. In a German hospital, VR-*E. faecium* (VR-Efm) rates increased from 11.2% (2014) to 26.1% (2017) [28]. Studies have demonstrated that enterococcal bloodstream infections (BSIs) caused by VRE are associated with a three-fold increase in 30-day mortality when effective treatment is delayed beyond 48 h [29]. Rapid molecular diagnostic tests have been shown to significantly reduce mortality risk and shorten hospital stays compared to traditional microbiological methods, which typically require 24–72 h [30]. Therefore, there is an urgent need to develop a rapid, and accurate detection method to simultaneously screen and monitor multiple resistance genes to last-resort antibiotics in the field more effectively.

In this study, end-functionalized primers were used to amplify target genes, enabling the integration of RPA technology with LFS via dsDNA amplicon bridging for the simultaneous detection of the VRE resistance genes *vanA*, *vanB*, and *vanM* in a single reaction. Our platform comprises three steps, namely DNA extraction, mRPA reaction, and LFS detection, and can be completed within 40 min (Fig. 1). Crude DNA was rapidly extracted using a Lysis Buffer, eliminating the purification step. This approach reduced processing time and minimized the risk of cross-contamination. The RPA amplification process is operable within a broad temperature range of 25°C to 45°C, allowing for the use of basic heating devices or ambient body heat to facilitate successful amplification. An operating temperature of 37°C and a reaction time of 20 min were selected to avoid non-specific amplification that may occur during prolonged incubation. Prior to LFS analysis, RPA amplicons were diluted in buffer at a 1:50 ratio to diminish background interference and prevent non-specific binding. Finally, the LFS results were interpreted within 5 min to prevent the development of false-positive signals. To validate the specificity of this method, 12 other common pathogens were tested, and no evidence of cross-reactivity was observed (Fig. 3). Importantly, the mRPA-LFS platform for detecting *vanA*, *vanB*, and *vanM* achieved sensitivities up to 10^2^ copies/μL (Fig. 3). To verify the accuracy of the developed methods, 30 clinical *Enterococcus* strains were compared with the conventional PCR method, with results showing 100% agreement between our method and the conventional PCR method (Fig. 4, Table 3). Among the 20 VRE isolates, 18 were identified as the *vanA* genotype, while the *vanB* and *vanM* genotypes were detected in only one isolate each. Consistent with current epidemiological trends, our results confirm the clinical dominance of *vanA*-positive strains. This is likely attributable to the genés location on the highly mobile Tn1546 transposon, which facilitates efficient inter-strain transmission. A strain carrying the *vanA* or *vanM* genotype typically exhibits resistance to both vancomycin and teicoplanin, whereas a strain carrying the *vanB* genotype is resistant to vancomycin but remains susceptible to teicoplanin. Furthermore, heteroresistance was observed in the identified *vanM*-type strain, where bacterial subpopulations with differing vancomycin susceptibility coexisted within the same culture. Conventional antimicrobial susceptibility testing often fails to detect these minor resistant subpopulations, creating a risk of treatment failure. In contrast, the mRPA-LFS system developed in this study directly targets the resistance genes, enabling reliable identification of such heteroresistant strains regardless of their phenotypic expression. Our multiplex assay allows for simultaneous amplification and discrimination of all three target genes in a single-tube reaction within 40 min, substantially reducing both sample volume and hands-on time. This efficiency provides a critical time advantage for implementing subsequent infection control measures. Conversely, non-multiplex methods can detect only a single target per reaction, so identifying the *vanA*, *vanB*, and *vanM* genes requires three separate procedures, which is time-consuming and labor-intensive. Additionally, the multiplex approach conserves reagents and consumables, which reduces the direct cost per test and minimizes laboratory waste, offering significant economic and environmental benefits. Compared to the qPCR method referenced in previous studies [31], our assay offers significant advantages, including rapid turnaround time, operational simplicity, and independence from sophisticated instrumentation, making it particularly suitable for POCT.

Although our detection method has significant advantages, it also has certain limitations. Firstly, the relatively small number of clinical samples used to validate the accuracy of this detection method, with only 30 samples participating in the test, may limit the universality and reliability of the results. Secondly, the current platform is designed to detect only the *vanA*, *vanB*, and *vanM* genotypes and does not cover other less prevalent types, such as *vanD* or *vanE*. Given the continuous evolution of global antimicrobial resistance, future work will focus on incorporating additional resistance gene targets and expanding the sample size and diversity to enhance the platform's comprehensiveness. Despite these limitations, a detection platform based on mRPA-LFS was successfully developed for the simultaneous detection of vancomycin resistance genes *vanA*, *vanB*, and *vanM* in *Enterococcus*, offering high sensitivity, specificity, operational simplicity, and low cost.

## Supplemental Materials

Supplementary data for this paper are available on-line only at http://jmb.or.kr.



## Figures and Tables

**Table 1:** Bacteria and viruses involved in this study.

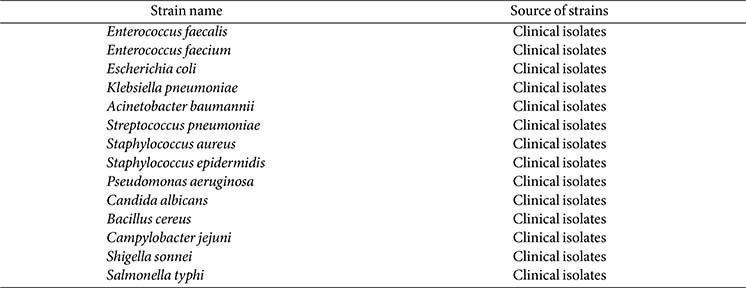

**Table 2:** Primers and probes sequences.

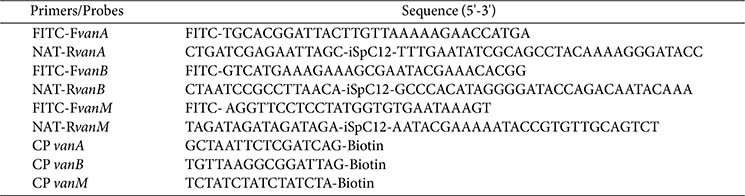

**Table 3:** Test results of 30 clinical samples.