# Supplementary material for: Simultaneous and Accurate Visual Detection of Vancomycin-Resistant Enterococci vanA, vanB and vanM by Multiplex Recombinase Polymerase Amplification Combined with Lateral Flow Strip
Source: J Microbiol Biotechnol. 2025 Nov 19;35:e2508037. doi: 10.4014/jmb.2508.08037 (PMC12640772; doi:10.4014/jmb.2508.08037)
Supplement: Supplementary file 1 [file jmb-35-e2508037-supple.pdf]

J. Microbiol. Biotechnol. 2025. 35: e2508037
https://doi.org/10.4014/jmb.2508.08037

Strain number	Gene types<br>vanA	 vanB	 vanM	Vancomycin MIC (μg/mL)	DNA concentration (ng/mL)
1	+	-	-	> 256	29.13
2	+	-	-	> 256	31.47
3	+	-	-	192	23.41
4	+	-	-	> 256	27.59
5	+	-	-	> 256	28.62
6	+	-	-	> 256	34.40
7	+	-	-	> 256	31.04
8	+	-	-	> 256	30.46
9	+	-	-	> 256	31.33
10	+	-	-	> 256	23.83
11	+	-	-	> 256	38.29
12	+	-	-	> 256	34.40
13	+	-	-	> 256	30.95
14	+	-	-	> 256	71.52
15	+	-	-	> 256	67.29
16	+	-	-	> 256	61.69.
17	+	-	-	> 256	50.92
18	+	-	-	> 256	65.23
19	-	+	-	64	21.89
20	-	-	+	> 256	64.23
21	-	-	-	1	38.94
22	-	-	-	1.5	45.25
23	-	-	-	0.75	49.56
24	-	-	-	1	61.36
25	-	-	-	0.75	66.08
26	-	-	-	0.5	47.04
27	-	-	-	1.5	35.59
28	-	-	-	0.75	41.04
29	-	-	-	2	25.78
30	-	-	-	0.5	52.78
